# Pulmonary toxoplasmosis in the immunocompetent

**DOI:** 10.11604/pamj.2025.50.104.47258

**Published:** 2025-04-22

**Authors:** Ashwin Karnan

**Affiliations:** 1 Department of Respiratory Medicine, Datta Meghe Institute of Higher Education and Research, Sawangi (Meghe), Wardha, Maharashtra, India

**Keywords:** Hemoptysis, lymphadenopathy, immunity

## Image in medicine

A 29-year-old male milk vendor, a non-smoker with no comorbid conditions, presented with complaints of fever, cough, hemoptysis, and loss of weight for the past 3 weeks. The complete blood count was normal, and all viral markers were negative. The chest X-ray was unremarkable, and the computed tomography (CT) scan of the thorax showed ground glass opacities in bilateral lower lobes with enlarged pre-vascular and pre-tracheal lymph nodes. Sputum for acid-fast bacilli was negative. Bronchoscopy was done, and transbronchial lung biopsy was taken from the right lower lobe, which showed cysts containing bradyzoites on histology, confirming the diagnosis of toxoplasmosis. Serum *Toxoplasma gondii* specific IgG titre was raised (23 IU/ml). The patient was started on tablet pyrimethamine 50mg/day and tablet clindamycin 900mg/day for 6 weeks. The patient is currently on follow-up and has shown symptomatic improvement. Pulmonary toxoplasmosis is usually a fatal protozoan infection caused by *Toxoplasma gondii* in immunocompromised individuals. The clinical features include cough, breathing difficulty, fever, hemoptysis, myalgia, arthralgia, and lymphadenopathy. Serological tests may sometimes be misleading, and a definitive diagnosis is usually made by bronchoalveolar lavage fluid polymerase chain reaction (BAL PCR) analysis, lung biopsy, and special staining with Gomori methenamine silver stain. Treatment options include pyrimethamine, sulfadiazine, sulfamethoxazole-trimethoprim, and clindamycin.

**Figure 1:**
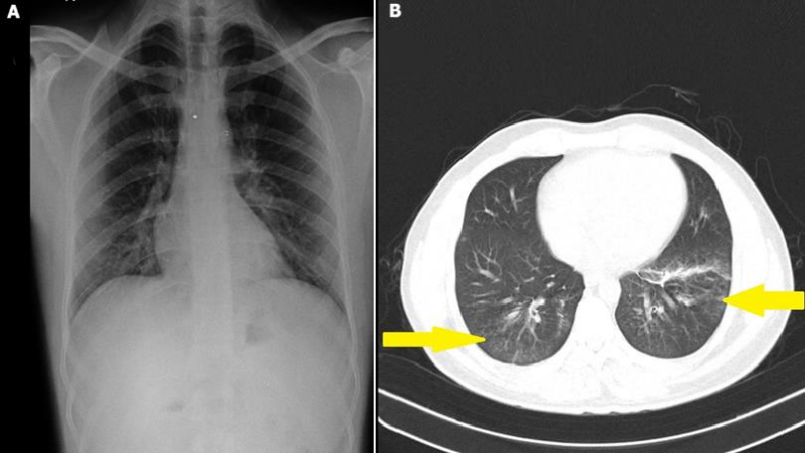
A) chest X-ray of the patient; B) computed tomography scan of the thorax showing bilateral lower lobe ground glass opacities

